# Humanity’s Best Friend: A Dog-Centric Approach to Addressing Global Challenges

**DOI:** 10.3390/ani10030502

**Published:** 2020-03-17

**Authors:** Naomi Sykes, Piers Beirne, Alexandra Horowitz, Ione Jones, Linda Kalof, Elinor Karlsson, Tammie King, Howard Litwak, Robbie A. McDonald, Luke John Murphy, Neil Pemberton, Daniel Promislow, Andrew Rowan, Peter W. Stahl, Jamshid Tehrani, Eric Tourigny, Clive D. L. Wynne, Eric Strauss, Greger Larson

**Affiliations:** 1 Department of Archaeology, University of Exeter, Exeter, Devon EX4 4QE, UK; N.Sykes@exeter.ac.uk; 2 Department of Criminology, University of Southern Maine, Portland, ME 04104, USA; beirne@maine.edu; 3 Department of Psychology, Barnard College, 3009 Broadway, New York, NY 10027, USA; ahorowit@barnard.edu; 4 Department of Math and Sciences, Exeter College, Exeter EX4 4HF, UK; ionewbjones@gmail.com; 5 Department of Sociology, Michigan State University, East Lansing, MI 48824, USA; lkalof@msu.edu; 6 Bioinformatics and Integrative Biology, University of Massachusetts Medical School, Worcester, MA 01605, USA; Elinor.Karlsson@umassmed.edu; 7 Broad Institute of MIT and Harvard, Cambridge, MA 02142, USA; 8 WALTHAM Petcare Science Institute, Waltham on the Wolds LE14 4RT, UK; Tammie.King@effem.com; 9 Annenberg PetSpace Foundation, 12005 Bluff Creek Dr, Playa Vista, CA 90094, USA; hdlitwak@gmail.com; 10 Environment and Sustainability Institute, University of Exeter, Penryn Campus, Penryn TR10 9FE, UK; R.McDonald@exeter.ac.uk; 11 Department of Archaeology, University of Iceland, 102 Reykjavík, Iceland; luke@luke-murphy.com; 12 Centre for the History of Science, Technology and Medicine (CHSTM), University of Manchester, Oxford Rd, Manchester M13 9PL, UK; neil.pemberton@manchester.ac.uk; 13 Department of Biology and Department of Pathology, University of Washington, Seattle, WA 98195, USA; promislo@uw.edu; 14 Wellbeing International, 9812 Falls Road #114-288, Potomac, MD 20854-3963, USA; arowan@wellbeingintl.org; 15 Department of Anthropology, University of Victoria, Victoria, BC V8W 2Y2, Canada; pstahl@uvic.ca; 16 Department of Anthropology, Durham University, Durham DH1 1LE, UK; jamie.tehrani@durham.ac.uk; 17 School of History, Classics and Archaeology, Newcastle University, Newcastle upon Tyne, NE1 7RU, UK; Eric.Tourigny@newcastle.ac.uk; 18 Department of Psychology, Arizona State University, Box 871104, Tempe, AZ 85281, USA; clivewynne@gmail.com; 19 LMU Center for Urban Resilience, Loyola Marymount University, LMU Drive Los Angeles, CA 90045-2659, USA; Eric.Strauss@lmu.edu; 20 Palaeogenomics & Bio-Archaeology Research Network, School of Archaeology, 1 South Parks Road, Oxford OX1 3TG, UK

**Keywords:** Strategic Development Goals, dog domestication, sustainable development

## Abstract

**Simple Summary:**

The Earth is under increasing pressure from the burgeoning global human population and the subsequent rise in demand for food and a myriad of other finite resources. Mitigating the environmental, societal and ecological impact of the human footprint requires understanding the long-term relationships between our species and the plants and animals we now rely upon. In addition, the modern scientific approach often conceives of, and addresses individual problems through narrow windows that can fail to take into account the connectedness of multiple problems. By broadening the scope of inquiry to include both science and humanities perspectives, and simultaneously focussing on a single species, we suggest that many of the United Nations Strategic Development Goals (SDGs) can be addressed more effectively. In this paper, we describe how a comprehensive assessment of the long-term relationship between humans and dogs can yield insights, and offer ways in which modern global challenges can be tackled.

**Abstract:**

No other animal has a closer mutualistic relationship with humans than the dog (*Canis familiaris*). Domesticated from the Eurasian grey wolf (*Canis lupus*), dogs have evolved alongside humans over millennia in a relationship that has transformed dogs and the environments in which humans and dogs have co-inhabited. The story of the dog is the story of recent humanity, in all its biological and cultural complexity. By exploring human-dog-environment interactions throughout time and space, it is possible not only to understand vital elements of global history, but also to critically assess our present-day relationship with the natural world, and to begin to mitigate future global challenges. In this paper, co-authored by researchers from across the natural and social sciences, arts and humanities, we argue that a dog-centric approach provides a new model for future academic enquiry and engagement with both the public and the global environmental agenda.

## 1. Introduction

Earth is under pressure from a growing human population and the associated intensification of food production, urbanisation, globalisation, inequality, conflict, environmental degradation and climate change. In 2015, following a global process of consultation, the United Nations launched *Transforming our World: the 2030 Agenda for Sustainable Development*. The agenda set out plans to tackle current global challenges through seventeen Sustainable Development Goals (SDGs) which span issues from food security and biodiversity conservation to health and education [1,2,3]. These interconnected SDGs were adopted by all UN member states in 2015 as a call to action to simultaneously reduce poverty and inequality while preserving natural resources and addressing climate change.

The 2030 Agenda for Sustainable Development, adopted by all United Nations Member States in 2015, provides a shared blueprint for peace and prosperity for people and the planet, now and into the future. At its heart are the 17 SDGs, which are an urgent call for action by all countries—developed and developing—in a global partnership. They recognize that ending poverty and other deprivations must go hand-in-hand with strategies that improve health and education, reduce inequality, and spur economic growth—all while tackling climate change and working to preserve the oceans and forests.

The importance of addressing the SDGs cannot be overstated, but the approaches through which long-term solutions might be achieved have been widely critiqued. Coming generally from the field of international development, which is itself the product of Western belief systems, strategies are often anthropocentric wherein humans are conceptualised either at the centre of the natural world, or as separate from it. As such, research agendas have prioritised narrowly focused scientific investigations, despite the fact that many global challenges are fundamentally cultural and possess deep histories. There is growing recognition that these global challenges will be better addressed if researchers and practitioners (from across the sciences, arts and humanities) work in concert to develop and apply new research methodologies and communicate their findings in accessible formats. 

Numerous interdisciplinary initiatives are already emerging. For instance, the One Health approach [4] recognises that human, animal, plant and environmental well-being are inextricably linked and cannot be understood, or managed in isolation. By coalescing broad efforts around specific challenges, One Health approaches have fostered transformative improvements in the mitigation of zoonotic diseases, as well as finding agricultural solutions to ameliorate hunger and reduce the negative impacts of global food production [4]. In spite of this progress, One Health is firmly set in the present, with little exploration of the historic and deeper time settings, contexts and mechanisms that have led to the emergence of modern global health challenges.

In this paper, we argue that a collaborative, interdisciplinary approach, united around a common theme, can successfully explore the dynamics and impacts of human-animal-environment interactions. An in-depth examination, focused initially on our relationship with just one other species, demonstrates not only the health impacts of these relationships, but also broader issues of global sustainability, social resilience and ecosystem management, while making research accessible to a wide variety of audiences.

As a demonstration of this approach, we focus on the domestic dog (*Canis familiaris*), a species that has held a closer relationship with humans, for a longer period of time, than any other domestic animal [5]. Humans, dogs, their mutualistic interactions and their respective societies, evolved alongside one another, sharing lives, diets, microbiomes, parasites and pathogens. The rich cultural history and diversity seen on the planet today was, in large part, co-created through human-animal relationships, and dogs were the first of our non-human relations to become intimate, impactful and durable. The success of human hunter-gatherers across at least five continents was often enhanced by the dogs they lived alongside (e.g., [6]). In addition, humans and dogs have maintained and extended their companionship, now globally ubiquitous, and both species have profited in extraordinary and diverse ways.

Dogs have always performed a variety of roles in human societies. We have made use of their skills and have asked them to perform an expanding range of novel jobs, from diagnosing human illnesses to lending the use of their senses to guide and protect human companions. This paper demonstrates how future prospects for the health and well-being of the human-dog-environment are inextricably linked. We argue that dogs may provide an even greater service to humanity; they can become an engaging medium through which innovative and interdisciplinary research findings focussed on global futures can be made accessible and communicated to a wide variety of audiences.

## 2. A Brief History of the Dog: Domestication and Dispersal

Descended from an as yet unidentified population of grey wolves [7,8], dogs were the first animals to enter into a domestic relationship with people. Despite (or perhaps because of) their longstanding history as a domestic animal, there is a great deal of uncertainty regarding the timing, circumstances, location and number of occasions on which dogs became domesticated [9]. What is clear is that dogs were integrated into human societies by at least 15,000 years ago, and that they began to occupy defined niches soon afterwards [5].

The early history of the human-dog relationship is probably best exemplified by how humans treated their dogs in death. Single dog burials have been excavated at hunter-gatherer sites at least ~10,000 years ago in the Americas [10], East Asia [11], the Near East [12] and in Europe [13]. The earliest human-dog co-burials date to 14,200 years ago [14], and there are numerous examples across Eurasia and the Americas reflecting the importance of the human-dog relationship.

Iconographic representations of dogs have a similarly deep history. The earliest suspected canid representations date to ~17,000–12,000 BP at the Grotte de Font-de-Gaume in France [15], and dog depictions are increasingly common after ~8000 years ago. For instance, dog images have been discovered on Arabian rock art at the site of Shuwaymis [16], with slightly later representations on Iranian pottery [17]. In many of these depictions, dogs are clearly shown as hunting companions. In ancient and indigenous mythologies, dogs are represented as trackers and guides (e.g., in Siberian and Amerindian tales), or as fearsome guards, such as the famous ‘hellhounds’ Cerberus of Ancient Greece and Sabala of Vedic tradition [18].

That dogs were primarily valued for their hunting or protective abilities is perhaps also reflected in the etymological origins of many widely-used terms for *Canis familiaris* which derive from the Proto-Indo-European root *kṷō̆n- or *k̑ū̌n- (‘sharp, fierce’) including: the Sanskrit *çvā́*, Greek *κύων*, Irish *cú* and Welsh *ci*, Latin *canis*, German *Hund*, and English *hound* [19,20,21]. With the exception of terms like the English term ‘*dog*’ and Spanish term ‘*perro*’ (both of which are of uncertain etymology), cross-cultural linguistic similarities suggest there may have been a common ancestor for many of the canine cultures of Western Eurasia. It has also been suggested, if not universally accepted, that other potential cognates are found in Uralic (*künjä*), Altaic (*qan/qin*) and Amerindian (e.g. Hokan *kwan* and Ge *okong*) languages [22].

These linguistic connections indicate a long-term correlation between specific human groups and their dogs, and recent studies of dogs have suggested that the history of genetically differentiated dog populations may mirror the human groups with which they were associated. For example, a recent study demonstrated that humans who arrived in Europe from the Near East ~8,000 years ago brought with them dogs who possessed a different genetic signature than those who were already associated with European hunter-gatherers [23]. A previous study [24] also showed a rise in the frequency of a different genetic type that may have been associated with the arrival of people with steppe ancestry during the Iron Age [25].

**Figure 1:**
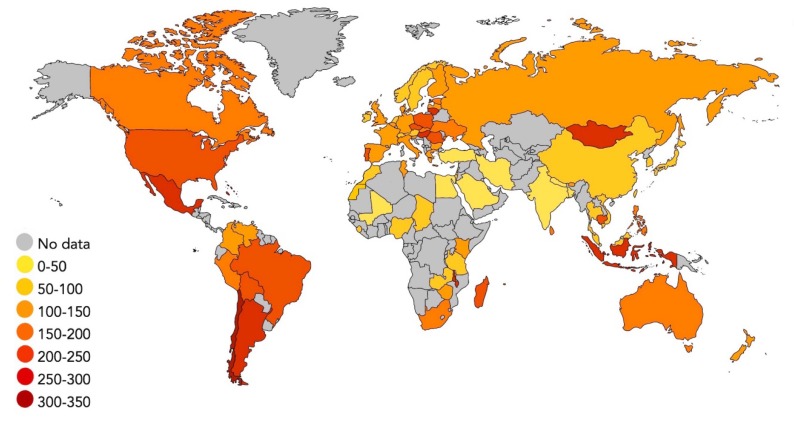
A map of countries across the globe depicting the frequency of dogs per 1,000 people. Darker shades represent countries with greater numbers of dogs per capita. Grey shading indicates countries for which data was not available.

Importantly, dogs were not a passive accompaniment to the expansion of people. Rather, they may have facilitated the movement of humans into new areas. For instance, a population of sled dogs and dog-related material culture on Zhokhov Island existed in the Siberian high Arctic 9000 years ago [26]. These dogs were closely related to the first dogs who entered the Americas alongside people, and may have been crucial for human migration across Beringia [27]. Dogs also dispersed with people across the world into Australia, the Pacific, South Africa, and South America, and by 1000 years ago dogs were present at the extremes of every inhabited continent [28]. Today, there are an estimated 700 million to 1 billion dogs across the globe [29,30]. Though the average global prevalence is ~120 dogs per 1000 people, dog density per capita varies considerably among continents and even between neighbouring countries (Figure 1).

Human-dog relationships are equally diverse. Dogs are viewed variously as deities, family members, workers, ‘street dogs’, anathema or food-stuffs. In addition, cultural values attached to dogs shift continuously in response to broader economic and ideological trends. For instance, though some of the earliest archaeological evidence for close human-dog relationships (e.g., individual burials, human-dog co-burials and iconographic representations) is present in the Middle East, this region today has some of the lowest numbers of dogs per capita (Figure 1). Long-standing religious taboos, alluded to in the early writings of many Semitic cultures (e.g., the Old Testament, Rabbinic literature, patristic documents and Ḥadīth), are frequently cited as the drivers for this transformation in human-dog relationships. However, it has been argued that animosity towards dogs—and street dogs in particular—developed more recently in the 1800s in response to new notions of urban sanitisation, hygiene and public health [31].

Dog numbers per capita are much higher across Europe and the United States, but in these countries, dogs are kept almost exclusively within the home, and street dogs have, with exceptions, decreased over the past fifty years [32]. These equally reflect long-term shifts in human-dog relationships. Archaeological and art historical studies, for example, reveal that the keeping of dogs as ‘pets’ or companions (as might be recognised in the West today) was present across ancient Mesopotamia, Egypt and the Greco-Roman world and eventually spread across the Roman Empire [33,34,35]. There is clear evidence that some dogs were treated as members of the family and were even interred in human cemeteries or given elaborate tombstones [34,35]. 

The concept of dogs as favoured human companions, however, appears to have dissolved across much of Europe with the collapse of the Roman Empire (AD 476), and only re-emerged in the Middle Ages [36,37,38,39,40]. At this time, as throughout most of history, pet-keeping was largely restricted to the social elite. It was not until the 17^th^ century AD, that dogs became companions to humans on a large scale, and only during the 19th century that dog-keeping became more widespread across social groups [41,42,43].

European history highlights that pet-keeping was intimately linked with economic prosperity. This association continues into the present with increasing prevalence around the world, as pet-keeping is increasing quickly within many developing countries. The popularity of companion dogs is even overcoming cultural taboos. For instance, there is a large and increasing population of pet dogs within Tel Aviv, Israel, even though traditional religious beliefs in the region are opposed to keeping pets in general, and dogs in particular [44]. 

A variety of terms have been used to describe these differing relationships. In a recent paper, an international consortium clarified the terminology used to classify dogs, based upon the closeness of their relationships with people [45]. Those dogs who have virtually no interaction with people are referred to as feral (e.g., dingoes) since, despite the fact that they descend from domestic dogs, they live primarily as wild animals. Positioned slightly closer to people and referred to as village or street dogs is the population that makes up the majority of the global population. These free-breeding and free-ranging dogs live alongside people and in rural and metropolitan areas, some of whom, for example in Chad, nevertheless have owners, names and addresses. Registered or purebred dogs, whose breeding and lives are the most controlled by people, occupy the last category. These dogs primarily descend from the Victorian breeding tradition [46], possess long-term pedigrees, and have been subjected to strong selection for behaviour, shape and colour. Purebred dogs have become increasingly popular throughout the world and in many northern countries are the dominant canine category.

Bringing together evidence from across the world and through time, demonstrates that there is no singular, transhistorical dog, and no one manner in which humans and dogs have related. Relationships with dogs are, and have always been, dynamic: materially, spatially, culturally and socially. Human-dog relationships are products of cultures, and their status in society has varied dramatically across different times and geographic regions of the world. As such, Figure 1 provides a low-resolution snapshot of what is a highly complex and nuanced dataset pertaining to human-dog cultural history. By paying attention to dogs and by exploring and narrating these relationships across diverse geographies, temporalities and materialities, we are sensitised to the wider ethical and political obligations that these relations demand, thus creating worlds that are less harmful to dogs and their human partners. 

## 3. Impact on Terrestrial Biodiversity (SDG 15: Life on Land)

In his pioneering work on the origins of animal domestication, Zeuner [47] described five stages of domestication, of which the last is the “persecution/extermination of wild ancestors”. His statement was made just two years before the International Union for the Conservation of Nature (IUCN) founded its Red List of Threatened Species. This was set up to document the threats to biodiversity and support the prevention of species extinctions, a fate that had already befallen the wild ancestors of many modern domestic animals including cattle, horses, and dromedary camels [48], and continues to threaten others including the grey wolf.

The IUCN states that the grey wolf’s original global range “has been reduced by about one-third, primarily in developed areas of Europe, Asia, Mexico and the United States by poisoning and deliberate persecution due to depredation on livestock.” Indeed, the species was extirpated in the British Isles, Japan and elsewhere, and although the timing and circumstances of these extirpations are uncertain, current hypotheses link the wolf’s demise, in part, to the rise of domestic dog populations [49]. In addition, conservation efforts to protect wolves or reintroduce them to the wild have been hampered by hostile attitudes that have been shaped by centuries of the fear of wolves (given their threat to livestock), and the resulting negative depictions of the ‘Big Bad Wolf’ in narratives and traditional folklore [50].

The geographic expansion of human populations and the consequent encroachment of domestic dogs is also accelerating the decline of other wild canid populations. After experiencing the detrimental effects of competition with expanding lion (*Panthera leo*) and hyaena (*Crocuta crocuta*) populations, African wild dogs (*Lycaon pictus*) went extinct in the Serengeti in 1991, at least partly as a result of epizootic canine distemper in sympatric domestic dogs [51,52,53]. In 2002, the captive breeding population in Tanzania was reduced from 52 to 3 by the same disease, again transmitted by domestic dogs [54]. Similarly, populations of the endangered Ethiopian wolf (*Canis simensis*) have been reduced by around 75% over the past 20 years due to dog-transmitted rabies epidemics [29]. Lembo [55] and colleagues have argued that dog-mediated rabies in humans could be eliminated throughout Africa, through a campaign of domestic dog vaccination. This approach would require a coordinated interdisciplinary effort that is currently in development.

In North America, dogs come into conflict with other synanthropic canid species, such as the coyote (*Canis latrans*), who is rapidly adapting to urbanised human communities [56]. Colonisation of urban habitats often places coyotes in direct competition with domestic dogs, causing significant management problems for city officials and pet owners alike [57]. A deeper understanding of the social and ecological connections between domestic dogs and coyotes could provide new insights into effective cohabitation among humans and domestic and wild canids. 

Considering domestic dogs from an ecological perspective provides an opportunity to evaluate their role in shaping the biodiversity and structure of human-dominated landscapes. For instance, dogs have been implicated in the decline of many threatened and now-extinct vertebrate fauna in Australia and New Zealand [29,58,59,60]. They are also impacting biodiversity on many Caribbean islands [61]. The logical inconsistency of human practices is evident in the response to hurricane Irma that struck the islands of Barbuda and Antigua in 2017. The limited international aid that was deployed for animals focused almost exclusively on saving the species—dogs and cats—responsible for the most damage to the islands’ native fauna [62].

At the same time, dogs can play an important role in conservation efforts. For instance, the dogs’ olfactory acuity and trainability are thought to enable them to find animals at rates often much higher than humans [63]. Through scat, carcass or direct detection of live or dead animals and their remains, trained dogs have been deployed to identify individuals and populations of birds, mammals and reptiles, from spotted and barred owls [64] to bobcat [65] and gecko [66]. Dogs have been successfully used to locate and/or identify numerous endangered populations such as the lowland gorilla [67] and the Northern Atlantic right whale [68]. Dogs also support the management of undesirable wildlife, particularly invasive, non-native species that are the target of management and invasion-prevention actions [69,70].

## 4. Human-Dog Diets (SDG 14: Life below Water; 13: Climate Action and 2: Zero Hunger)

Wolves and people likely did not enter into their relationship through a deliberate, human-initiated process. Instead, wolves followed what is referred to as a commensal pathway [71,72]. This trajectory involved a multi-stage acclimation of wild wolves to humans and human niches, beginning with an “anthropophily” stage that allowed wolves and people to establish the foundations of a more direct and reciprocal relationship. Within Zeder’s [71] model, the transition from a synanthropic wolf population to a domestic dog took place only after wolves progressed from anthropophily to habituation, to commensalism and partnership. A final stage proposed by Zeder, captivity and human controlled breeding, is only true for a minority of dogs, and is primarily a recent phenomenon. 

Evidence for this shift in the intensity of human-dog relationships can be seen in analyses of stable isotopes derived from archaeological dog remains. More specifically, a study of isotopic values derived from early Chinese dogs [73] concluded that the canids on the site were more likely dogs since their dietary values mirrored that of the humans and suggested they were consuming nearly identical diets. This isotopic similarity reflects the shift toward commensalism as dogs became more reliant on people for their sustenance. 

**Figure 2:**
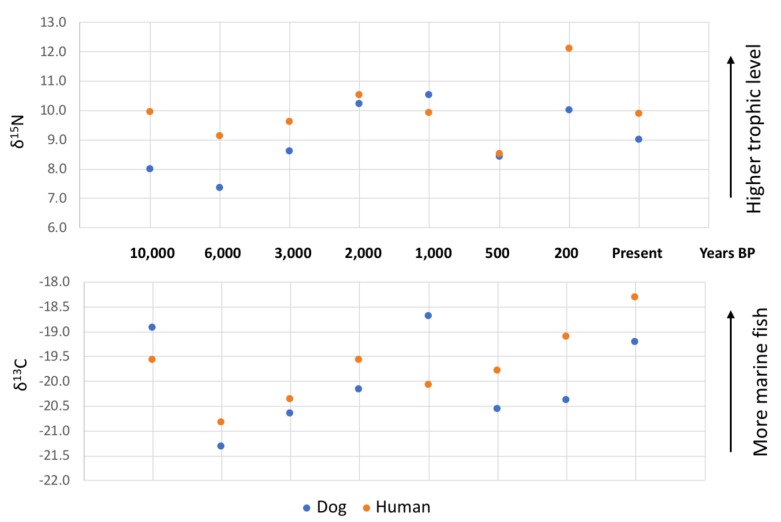
A diagram demonstrating the long-term history of the shifting isotopic values that reflect human and dog diets in Britain over the last 10,000 years. The parallel shifts in the carbon and nitrogen values between both species suggests a closeness of the human-dog relationship over the past 10 millennia [75].

Isotope studies with a long-term perspective have demonstrated that human and dog diets have moved in parallel throughout time [74,75]. For instance, longitudinal studies from Britain (Figure 2) show that in periods when humans were consuming a diet comprised of relatively greater quantities of animal-derived proteins, (indicated by enriched δ^15^N values) the same was true for dogs. Similarly, periods of increased consumption of marine-derived nutrients, likely sea fish, can be seen in the similarly enriched δ^13^C values of both humans and dogs. This dietary mirroring is perhaps unsurprising, given that, for most of human history and in the majority of cultures today, dogs have subsisted off human leftovers and/or faeces. It was not until the 1860s that the concept of specially manufactured ‘dog food’ emerged. The idea developed in Britain and was soon taken up in the US. Initially, dog food was expensive and only purchased by people involved in dog shows or the breeding of hunting dogs [76]. 

Over the past one hundred years, the intensification of pet-keeping has seen a commensurate rise in the development of foodstuffs tailored to pets and, more specifically, to the age, health, breed and size of pet dogs. However, feeding practices are often heavily influenced by owner perceptions about what is ‘healthy’ in human terms, or even trendy. For example, a company called Fish4Dogs markets food and treats for dogs on the assumption that fish is a superior source of protein. In addition, the rise of the ‘palaeodiet’ (the belief that humans should eat high protein, low carbohydrate foods more akin to the fare of prehistoric hunter-gatherers) has been accompanied by the appearance of ‘grain-free’ dog-foods, marketed as more appropriate for the dog’s lupine ancestry [77]. Following more recent food trends, several brands of pumpkin spice latte dog food are also available.

Crucially, domestic dogs and modern wolves have evolved different physiologies over the past 15,000 years. Many dogs carry genetic changes that mirror genetic adaptations in humans that allow us to extract more energy from a starch-rich diet [78,79,80]. These evolutionary changes associated with an adaptation to living in close proximity to people suggests that, even though modern wolves and dogs share a recent common ancestor, a lupine diet may not necessarily be ideal for dogs. Leaving aside the potential issues associated with grain-free diets, inappropriate feeding is a major problem and one of the most common veterinary interventions is diet-related tooth removal. For instance, Wallis Annenberg PetSpace Foundation reports that approximately 70% of the dogs they take in for adoption require one or more veterinary procedures. Although spay/neuter surgery is the most prevalent, the second most common at 16% is dental procedures for advanced periodontal disease (personal communication, J.J. Rawlinson, DVM). 

Feeding decisions also have environmental consequences. For instance, the overfishing of marine pelagic fish stocks is being driven not just by human consumption, but also by demands for pet food. Figure 2 demonstrates the likely increasing contribution of marine fish to dog diet over the last 500 years and, over the last few decades, demands have risen further. While in 1970 0.9 million tons of fish were converted into pet-food, that amount in 2006 had risen to 13.1 million tons [81,82].

The impact of the pet food industry is also felt in terms of terrestrial resource use, as its production requires large quantities of land, water and fossil fuels. In the United States, it is estimated that dogs and cats contributed an additional 25%–30% of the environmental impact wrought by humans and are responsible for release of up to 64 million tons of greenhouse gasses [83]. In China, it is estimated that dogs and cats use resources equivalent to 70–245 million people, producing a carbon footprint equivalent to 34–107 million people [84]. As pet ownership increases in developing countries, it is anticipated that the impact of dogs and cats will add significantly to issues of global food security and environmental degradation [81,83,84,85,86].

Aside from its environmental impact, there are ethical issues associated with feeding dogs quality protein that could be used to feed the estimated 925 million people worldwide who are hungry or malnourished [87]. In some developing countries, where high levels of malnutrition exist, dogs themselves are considered a source of food, and their meat is praised for its taste and medicinal qualities [88,89]. Dog-eating communities around the world have come under intense scrutiny and criticism from Western cultures. In most cases, Western influence has suppressed or eradicated dog-meat consumption, often encouraging its replacement with more Western food practices [90]. However, rates of meat consumption are much higher in Western cultures, with far greater levels of environmental impact, than are seen in developing countries. Notably, consumption of meat in South Korea, where dogs are still eaten, averages 42 kg per capita per year compared to 76 kg in the UK and 124kg in the US. In Niger, where dogs are also occasionally consumed, meat consumption is just 8.1 kg [91].

Hunger is just one side of the double burden of malnutrition. The other is obesity. The World Health Organisation highlights that the health consequences of over-consumption are now as serious as those associated with starvation, if not more so. An estimated 1.9 billion people are thought to be overweight and the majority of these come from developed countries with the highest rates of meat consumption, notably the US and within Europe. It is also within these countries that dog obesity is now on the rise [92], perhaps unsurprisingly given the long-term association between human-dog diet and health. In recognition of these links between human and dog well-being, there are increasing calls for obesity to be seen as a One Health issue. Collaborations between human and veterinary medicine specialists could address not only dietary links, but also a range of additional health issues shared by humans and dogs.

## 5. Human-Dog Life and Death (SDG 3: Good Health and Well-being)

Of all the domestic animals, dogs share the greatest number of known pathogens with humans. It has been demonstrated in [93] that this is correlated with the duration of the domestic relationship: dogs host approximately 30 diseases and parasites transmissible to humans while cats and pigeons only share ten and seven pathogens respectively. Transmission of pathogens is frequently cited as the driver of widespread cultural taboos against dogs, and it is generally assumed that this enmity resulted particularly from dogs as a source of rabies infection [94]. On the basis of historical evidence, rabies may have been recorded in Middle Eastern documents dating to 2,300 BCE [95], and the disease reached Europe after the 5^th^ century BCE [96], though retrospective diagnosis of diseases on the basis of historical records can be fallible [97,98].

Regardless of whether rabies in dogs was the driver of dog taboos, the disease is currently a major health issue for both dogs and humans in India, and many other areas of Asia and Africa [99]. Understanding dog–human relationships in communities is critical for planning rabies prevention and control. Certainly free-breeding dogs (though they are still ‘owned’) constitute the major component of the rabies threat and represent a public health issue, especially in developing countries. Such dogs are estimated to number ~500 million, which could be almost 50% of the global dog population [32]. 

Dogs do not need to be rabid for their bites to pose a significant health problem. Though the values lack precision, there are approximately 450,000 dog bites treated in medical emergency rooms in the USA each year [100], and fatality rates are higher in less wealthy countries [101]. Within Western countries, concerted efforts have been made to eradicate all free-breeding dogs. Starting in the nineteenth century, the first modern home for stray dogs—London’s Battersea Dogs Home—was established in 1860 primarily to control rabies and to limit stray dog populations by euthanasia [76].

More recently, amongst the most contentious public health issues in the West has related to dog faeces. In 1970s Britain, excrement became the target of the Children Before Dogs movement, and in 1978, New York’s mayor Ed Koch introduced the so-called “poop scoop” law that has become the dominant approach for addressing issues of environmental hygiene around dog excrement in urban areas. 

As a consequence of a global eradication campaign, the global incidence of the debilitating nematode parasite Guinea worm (*Dracunculus medinensis*) in humans dropped dramatically from ~3.5 million per annum in the 1980s, to 28 in 2018 [102]. Just as eradication appeared imminent, however, the 2010s saw the apparent emergence of a reservoir of Guinea worm infection in domestic dogs. In 2018 in Chad, the worst affected country, there were 1040 dogs with 2044 emergent worms, and only 17 human cases [102]. The worms in dogs and people are genetically indistinguishable [103], highlighting the close interrelationship of human and dog health. Dog infections now represent the major impediment to global eradication of this human disease and the pathogen that causes it. Rapid development of new tools for diagnosis (serology) and control in dogs (anthelmintics) is being undertaken to complement the further intensified application of controls (surveillance, case isolation, water treatment) that had proven so effective for controlling Guinea worm infections in people.

The close relationship of dogs and humans, while driving some of the negative health impacts of dogs, also offers opportunities to discover new therapeutic avenues for treating common diseases with high morbidity and mortality. Dogs suffer from many of the same diseases as humans, including cancers, epilepsy, diabetes, arthritis, and psychiatric disorders. Just like humans, dogs develop these diseases naturally, over the course of their lives, as a result of both environmental and genetic factors. Dogs show the same age-related decline in function that we experience, share a common environment, and are provided a health care system second only in sophistication to our own [104,105]. Finally, the very large size of the dog population means that powerful genomic studies to find disease risk factors are feasible [106]. 

Since the publication of the dog genome [7], researchers have focused on finding the genetic factors influencing disease risk in purebred dogs. Each purebred dog breed, which is a genetically isolated population with relatively limited diversity, suffers from its own constellation of particular diseases. Where some breeds commonly suffer from heart disease (e.g., Chihuahuas), bone cancer (e.g., Great Danes), or respiratory illness (e.g., French Bulldogs), other breeds rarely experience these specific diseases, but instead show their own unique set of health risks. Because dogs within a breed are closely related, it is easier to find the genetic changes associated with these increased risks, and, for some single gene disorders, the precise disease-causing mutation [107]. One of the first genetic insights into narcolepsy, a sleep disorder affecting both dogs and humans [107], was the discovery of a mutation in a hypocretin receptor gene in affected Doberman Pinscher dogs [108]. This discovery has since led to critical discoveries about the molecular biology of sleep [109,110] and new treatment options for people suffering from this disease [111].

The potential power of dogs for advancing healthcare is much broader than genetic studies of dog breeds. Companion animals, including dogs, offer a unique opportunity to study the full range of complex factors—both environmental and biological—affecting both disease-risk and healthy aging. One of the major challenges in studying complex traits is the need for very large sample sizes, with detailed information on each individual and its environment. Ideally, such information should be collected longitudinally over the lifetime of the individual. Dogs are ideally suited for such research [112]. Most of the tens of millions of dogs in the US and other high-income nations have a human family that monitors their health and wellbeing, and will care for them into old age. As such, the human-dog family become de facto study teams of researcher and subject that can both enrich the knowledge to the scientific community and enhance the participation by the human community in citizen science [113]. Efforts to establish usable databases for the epidemiological study of canine disease have been marked by fifty years of failure, though VetCompass, an initiative by the Royal College of Veterinary Surgeons in the UK and veterinarians in Australia may finally begin to be able to study the epidemiology of canine diseases.

Dogs assist human health and well-being in more practical ways as well. For nearly a century, dogs have been providing support and assistance to vulnerable human populations, such as guide dogs for the blind and hearing dogs for the deaf [114]. More recently, there has been a rise in service dogs trained to work with other specialized populations, such as war veterans suffering from post-traumatic stress disorder [115]. The last few decades have seen the growth of detection dogs, the majority of whom are used in law-enforcement purposes. Increasingly, dogs have been employed in health-oriented detection businesses, including those specialising in detection of bed-bugs [116]. A number of researchers have trained dogs to detect cancers including: bladder [117], lungs [118], prostate [119], breast [120] and skin [121], as well as hypoglycemic and hyperglycemic episodes in diabetics [122] and epileptic seizures [123]. In addition, pet dogs can provide not only companionship, but also therapy to groups of people in hospitals, aged care homes, schools and more recently, airports and universities [124]. 

Many studies have assessed the benefits pets bring to people and a review of the literature shows many potential associations. Children who grow up with pets may have higher self-esteem, more empathy, are more popular with their peers, are more involved in hobbies and chores, experience fewer allergies and less asthma, have fewer sick days from school and are less likely to be obese relative to children who do not have a pet [125]. On the opposite end of the age spectrum, one study found that older adults who have pets experience increased mental function, are more social, are physically more active, have lower blood pressure and experience less stress, loneliness and depression, compared to those who do not have pets [126]. Another study, however, found that walking leashed dogs increased fracture risk in older adults [127]. A comprehensive quantitative assessment of the risks and benefits of dog ownership has not yet been confidently established, since this research field is still in its infancy [128].

As dogs come to be viewed increasingly as family members across much of the developed world, the grief felt at the loss of a beloved dog can be significant [129,130,131]. Veterinary and animal-shelter industry professionals are particularly at risk since they routinely encounter work-related stressors caring for companion animals, often resulting in occupational stress and burnout, mental health problems, as well as compassion fatigue and suicide [132]. In order to cater for the growing pet population, recognition of the problems faced by these people, along with support initiatives, are essential to ensure job retention and satisfaction in professions that care for companion animals. Investigating these topics also provides an opportunity to consider both human and animal end of life care. Thinking about people and their pets in this regard in parallel provides an opportunity to explore these questions in a context which, for many, is emotionally safer and easier. In this way, dogs could regain one of their original roles in ancient societies, as psychopomps who guided human souls to the underworld [133].

## 6. Conclusions

An investigation of dogs and the human-dog bond has generated impressive insights into the underlying mechanisms of diseases, aging and social resilience. These complex issues encompass the entire endeavour of human intellectual inquiry including: biology, veterinary sciences, history, art, ethics, technology, economics, sociology and politics, to name just a few. More than simply objects of academic investigation, we argue that understanding the unique breadth and depth of the evolution, impact and dynamics of human-dog relationships can lead to novel insights when addressing the global challenges that humans and dogs have co-created. Others have adopted a similar perspective. For instance, a recent review of domestic cats called for the emphasis to be placed on the breadth of roles they play within human societies [134], while another review [135] demonstrated the health and environmental consequences of the relationships between humans and domesticated animals. The approach outlined here is a case study for a novel model of examining the ecology of humans, not in the traditional sense as an isolated species of ecosystem engineers, but embedded in relationships with domesticated animals who serve as an impact multiplier.

Perhaps not surprisingly, numerous academic disciplines make use of dogs to gain insights into their respective fields. Table 1 presents a snapshot of how dogs are used, and what insights they can lead to in a wide variety of unrelated disciplines. Because dogs cut across so many disparate academic pursuits, they can easily be used as a means to focus the attention of students as case studies for exemplifying key concepts. More specifically, studies of dogs can lead to the development of new animal-based research model systems for understanding human wellness across multiple scales, and the use of the human-animal bond to inform educational strategies across the spectrum of ages and learning capabilities. 

The emergence of the evolutionary, economic and social relationships between humans and dogs further serves as an example of the constellation of relationships that exist between humans and all other domestic and wild animals. Effectively tackling modern challenges exemplified by the SDGs requires a perspective that not only places humans within the animal kingdom, but also considers the deep time and shifting relationships between people and all other organisms. Focusing on relationships, and thinking about the degree of synanthropy of each species in space and time forces us to consider the cultural context and variability of those relationships. This more aware, and contextually-embedded approach will not only further our understanding of the complexity of these relationships, it will create an opportunity to establish new models for understanding and elucidating scientific questions and research models. On a more practical level, this approach will also allow for a more sophisticated appreciation of the issues that we face and lead to more effective strategies for addressing the major global challenges.

## Figures and Tables

**Table 1:** The potential and realized benefits of dog-focussed studies across multiple disciplines.

Discipline	Types of Studies That Involve Dogs	Benefits and Potential Insights
Cognitive Psychology	Humans identify themselves within the animal kingdom.	By placing humans within nature, dog studies engage biophilia and can improve human psychological health.
Human Medicine	Dogs as models for understanding the acquisition and progression of human disease (especially genetic and degenerative diseases).	Enhances the role of the human-dog interaction as a tool for understanding environmental factors in medicine and improves public health and opportunities for novel research models.
Ecology and Evolution	Study model for domestication, natural and artificial selection. Companion animal ecology. Ecological interactions among wild and domestic species. Detection of species of conservation interest.	Better understanding of evolution, selective pressures and adaptive mechanisms across all species, and an improved basis for human animal management. Improved management of wildlife.
Restorative Sociology	Domestic dogs as models for human socialization from trauma and injury.	Improved recovery of humans from traumatic physical and psychological injury.
Learning Sciences	Human-animal bond as a model for teaching both science, technology, engineering and mathematics (STEM), and arts and writing. Dogs as reading partners for beginning learners.	Provides focus and models for curricula in science and engineering and improves learning outcomes and jobs readiness, enhances STEM career preparedness especially in animal-based careers.
Immunology	Role of early exposure to pets in boosting immune response.	Canine model provides easy access to permanent human-animal bonds in the household and improves health outcomes in people.
Pain Science	Human-dog engagement and the resulting analgesics in pain management.	Reduced use of pre-and postoperative pain medications, especially in paediatric applications and reduced dependency on opioid and other addictive analgesic medications.
Behavioural Neuroscience	Role of human-dog contact in social skill development for autism-spectrum patients.	Serves as a novel model for behavioural development, especially in domestic-hybrid models such as dogs and wolves and increased socialization capacity for children with autism-spectrum disorder, and possible development of novel therapies.
